# Supplementary material for: Identification and functional analysis of non-coding regulatory small RNA FenSr3 in Bacillus amyloliquefaciens LPB-18
Source: PeerJ. 2023 May 15;11:e15236. doi: 10.7717/peerj.15236 (PMC10194069; doi:10.7717/peerj.15236)
Supplement: Supplemental Information 4 [file peerj-11-15236-s004.zip › KO/CK-vs-T1_map/map07222.html]

KEGG PATHWAY: Peroxisome proliferator-activated receptor (PPAR) agonists - Reference pathway


|  |  |
| --- | --- |
| **Peroxisome proliferator-activated receptor (PPAR) agonists - Reference pathway** |  |

|  |  |  |
| --- | --- | --- |
| [ Pathway menu \| Pathway entry \| User data mapping ] | 184% 150% 122% 100% 82% 67% 55% | 图片下载 |
